# Near-Infrared Laser-Assisted Indocyanine Green Imaging for Optimizing the Design of the Anterolateral Thigh Flap

**Published:** 2012-07-05

**Authors:** Justin M. Sacks, Alexander T. Nguyen, Justin M. Broyles, Peirong Yu, Ian L. Valerio, Donald P. Baumann

**Affiliations:** ^a^Department of Plastic and Reconstructive Surgery, The Johns Hopkins School of Medicine, Baltimore, MD; ^b^Department of Plastic Surgery, The University of Texas MD Anderson Cancer Center, Houston, TX; ^c^Department of Plastic and Reconstructive Surgery, Walter Reed National Military Medical Center, Bethesda, MD. Justin M. Sacks, MD, is a consultant and speaker for LifeCell

## Abstract

**Objective:** The anterolateral thigh flap is a versatile flap that can be used in a free or pedicled fashion. Because of the large amount of potential soft tissue, low donor site morbidity, and long pedicle lengths, many researchers consider it to be the perfect free flap. However, dissection of this and other perforator flaps can become an arduous experience with learning curves to overcome. Near-infrared laser angiography using indocyanine green provides a useful adjunctive tool to more predictably assess direct perforator perfusion zones. Laser-assisted angiography with SPY-Q analysis gives live localization of the flap's dominant perforator perfusion zones while quantifying the relative tissue perfusion for immediate skin paddle design. **Methods:** Fifteen patients with head and neck cancer defects were reconstructed with a free anterolateral thigh flap using laser-assisted near-infrared indocyanine green perforator mapping. The mid-point of a line between the anterior superior iliac spine and the patella was determined and the laser was centered over this. Indocyanine green (12.5 mg) was injected intravenously and fluorescence patterns were recorded. Optimal perforators were chosen using real-time imaging and SPY-Q analysis software. The anterolateral thigh skin paddle was centered over perforators based on best relative perfusion values. The hand-held Doppler was not used to identify perforators. All flaps were elevated in standard fashion. Patient demographics, defect characteristics, reconstructive techniques, and clinical outcomes were assessed. **Results:** All 15 free flaps were raised with the assistance of laser-assisted angiography. Cutaneous Doppler did not aid in the design of the skin paddle. There was only 1 flap loss due to venous congestion. All donor defects were closed primarily without the need for a skin graft. **Conclusions:** Laser-assisted indocyanine green angiography using SPY-Q analysis software provides robust, intraoperative, objective data to optimize anterolateral thigh skin paddle design while potentially minimizing patient morbidity. Future studies will be needed to further evaluate the use of this new technology.

The anterolateral thigh (ALT) flap is a fasciocutaneous perforator flap with numerous advantages for free tissue reconstruction. While the ABC perforator system can assist with flap design, the ALT flap can still be difficult to design and harvest secondary to variable perforator anatomy.^1^ Although cutaneous Doppler examination can help in identifying the location of audible perforators, this method does not always reliably orient the cutaneous surface over the areas of maximal perfusion. These intricacies in ALT flap design are especially pertinent when making the initial anterior incision, which may or may not incorporate the necessary perforators. Thus, a novel system to accurately visualize perforators is needed to optimize flap design.

Near-infrared laser angiography using indocyanine green (ICG), a water-soluble tricarbocyanine dye, provides a useful adjunctive tool to more predictably assess the direct perforator perfusion zones. It also allows for a dynamic, real-time evaluation of flap perfusion. ICG contains less than 5% sodium iodine and is contraindicated in patients with iodides or iodinated contrast allergies. Indocyanine green absorbs light in the near-infrared spectrum. It binds to plasma proteins with a peak absorption at 800 to 810 nm. The half-life of ICG is 3 to 5 minutes and it is metabolized hepatically and excreted renally. With the use of multiple injections, the dose should not exceed 1 mg/kg.^2^

The SPY system (Novadaq Technologies, Inc, Concord, Ontario, Canada) was originally developed to evaluate patency of coronary artery bypass grafts, with the approval of the Food and Drug Administration expanded to plastic surgical procedures in 2007. For breast reconstruction, the SPY system has been proposed to aid in perforator identification, evaluation of tissue perfusion, and predicting potential regions of tissue necrosis.^2^^,^^3^ The SPY-Q analysis program can quantify relative and absolute tissue perfusion parameters based on the absorption patterns of light emitted from ICG, which is directly bound to intravascular proteins within the near-infrared spectrum.^4^

Laser-assisted ICG angiography is a modality that visualizes skin perfusion to flaps. This technology allows one to quantify tissue perforator perfusion zones in a dynamic and real-time application tool. In this series of reported cases, we will outline how to utilize ICG imaging to optimize the placement of the ALT flap skin paddle. This tool can not only reduce the incidence of poorly designed fasciocutaneous flaps by maximizing flap perfusion zones, but, also preclude the need for other preoperative adjuncts that may be more costly and less efficient.

## METHODS

Fifteen patients with head and neck cancer were reconstructed with ALT flaps using laser-assisted ICG perforator perfusion zone mapping. Patient demographics, defect characteristics, reconstructive techniques, and clinical outcomes were assessed. We retrospectively reviewed prospectively entered data from the computerized database in the Department of Plastic Surgery at the MD Anderson Cancer Center for all patients included in the study. This study was approved by the Institutional review board at The University of Texas MD Anderson Cancer Center.

**Figure 1:**
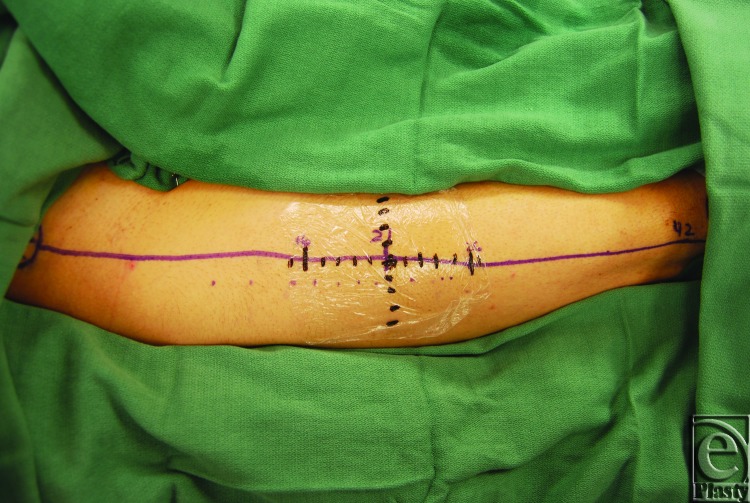
Donor leg displaying ABC perforator system with onlay XY grid.

For each patient, a line was drawn from the anterior superior iliac spine to the lateral border of the patella and the laser was centered over the midpoint as previously described.^5^ An X-Y system of one-centimeter hatch marks, centered on the midpoint, was drawn onto the leg using a marking pen (Fig 1). Indocyanine green (12.5 mg) was injected intravenously and live fluorescence patterns were recorded using near-infrared laser angiography.

**Figure 2:**
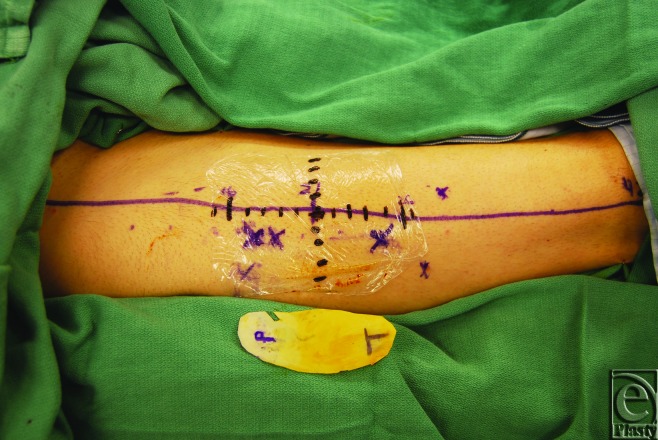
Donor leg displaying ABC perforator system with recipient site specifications drawn onto a template.

**Figure 3:**
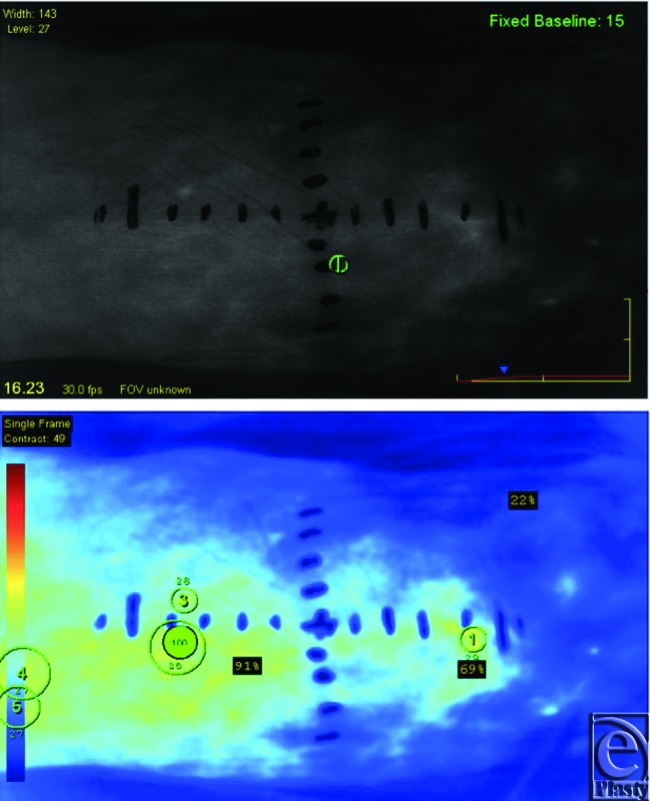
Intraoperative SPY Q analysis photo displaying zones of maximal perfusion corresponding points drawn on the XY grid system.

**Figure 4:**
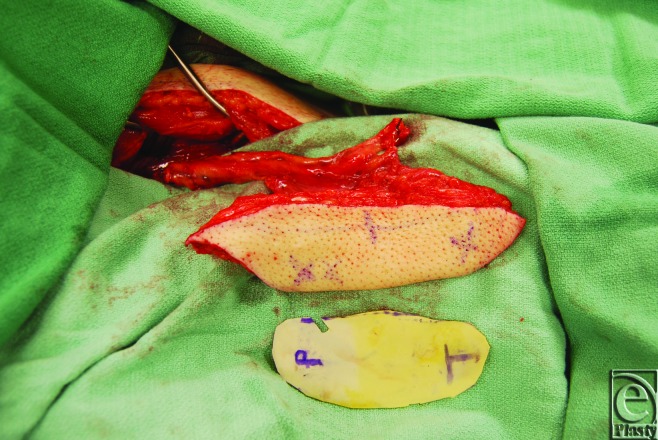
Elevation of the ALT flap over the zones of maximal perfusion. ALT indicates anterolateral thigh.

Optimal perforator perfusion zones were chosen using real-time imaging and SPY-Q analysis software. A template of the residual oncologic defect was made using an Esmarch dressing (Fig 2). Using the template, the skin paddle was centered over optimal perforator perfusion zones (Fig 3). All flaps were elevated in standard fashion (Fig 4). Handheld Doppler was not used to identify perforator signals preoperatively in aiding with the design of the flap.

## RESULTS

**Figure 5:**
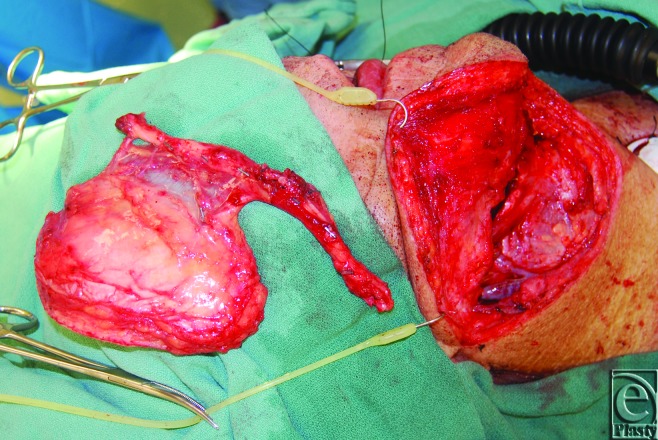
Inset of the ALT flap over large head and neck defect.

**Figure 6:**
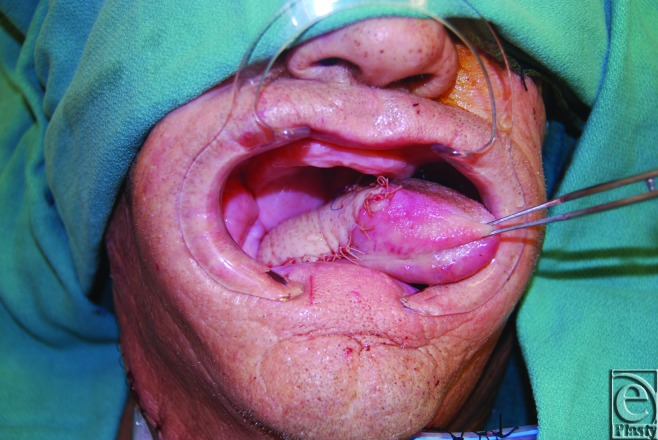
Resultant photograph showing reconstruction of the tongue and check with free ALT flap. ALT indicates anterolateral thigh.

Fifteen patients underwent free ALT tissue transfers for head and neck cancer reconstruction. (Figs 5 and 6) Intraoperative imaging using ICG assisted in placement of the ALT skin paddle over 1 or 2 optimal perforators for each flap were recorded and analyzed (Video 1). In this series, there were no complications related to the administration of ICG fluorescent dye. There was one flap loss secondary to venous thrombosis requiring debridement. All ALT donor sites were able to be closed primarily, with no other reconstructive measures such as skin grafting required.

## DISCUSSION

The ALT flap is both reliable and versatile, with relatively low donor-site morbidity.^6^ Because of perforator variability, capture of the axially based perforasome can be difficult if the skin paddle is not centered correctly.^7^ In addition, anatomic variations and perforator variability can lead to lengthy and arduous dissections, which may compromise flap perfusion and predispose to flap loss.^8^ Laser-assisted angiography can aid in optimizing flap design while decreasing donor site morbidity by incorporating maximal flap perfusion patterns in a real-time setting.

Other accepted preoperative design methodologies have their own limitations. A handheld unidirectional Doppler probe, while highly sensitive, frequently locates small perforators that may be insufficient to support a flap.^9^ In addition, sensitivity may also vary significantly with differential skin thickness.^5^^,^^10^ Computed tomographic angiography provides information as to the largest perforator to a specific area, but it does not provide dynamic information as to the actual perforator perfusion zones. In addition, there is a radiation and contrast load when using this assessment tool.

Laser-assisted angiography with SPY-Q analysis gives live localization of the flap's dominant perforator perfusion zones while quantifying the relative tissue perfusion for immediate skin paddle design. Using the SPY-Q analysis program, we were able to identify the dominant perforator perfusion zone on which to center our skin paddles. Our grid system further augments the ease of pinpointing these perfusion zones because one can objectively find this zone on the analysis image and correlate it directly within the grid located on the patient's donor thigh.

Dominant perforators may not travel directly to the dermis as shown in experimental studies of primary ALT flap thinning.^11^ Although dermal perfusion has been shown to correlate with actual perforators in a swine model, they may not directly map the perforators, which may be more noticeable in larger thighs where perforators may take indirect paths before reaching the subdermal plexus.^12^ Our method does not identify if a multipaddle ALT can be raised because the perforators are not directly visualized; however, this can be utilized after flap elevation to assess viability of multiple paddles prior to separation.

Our intraoperative angiographic analysis not only optimizes the planning and dissection of the ALT flap but also can provide valuable information about the viability of a prospective flap. Thus, if the perforators are not visible on one thigh, the operation can be aborted and the contralateral thigh may be used. This could potentially avoid unnecessary flap dissection. In the rare instance that neither flap is available for coverage, the operation can be aborted and other reconstructive options can be pursued.

The ALT flap can be raised successfully without using laser-assisted angiography but this can be at the expense of potentially harvesting a much larger skin paddle than required. By making the anterior incision more medially, the surgeon will increase the odds of capturing the septal musculocutaneous perforators. However, this will increase the likelihood of a difficult closure. We believe ICG analysis may be especially useful for less experienced surgeons in their flap design. We contend that the analysis provides valuable data about perforator perfusion zones and should be considered when designing the skin paddle. A larger experience using this technique will determine whether flap viability is actually improved by this method.

## CONCLUSIONS

Laser-assisted ICG angiography using SPY-Q analysis software provides robust, intraoperative, objective data to optimize ALT skin paddle design while potentially minimizing patient morbidity. Our grid system augments the ability to pinpoint the best zone of perforator perfusion. This imaging system has the potential to revolutionize and simplify the intraoperative design of perforator flaps.

## Figures and Tables

**Video 1.** Video detailing the use of the SPY system in designing an individualized free ALT flap. ALT indicates anterolateral thigh. [Click Here to view video]
